# Gait speed and handgrip strength as predictors of all-cause mortality and cardiovascular events in hemodialysis patients

**DOI:** 10.1186/s12882-020-01831-8

**Published:** 2020-05-06

**Authors:** Yu Ho Lee, Jin Sug Kim, Su-Woong Jung, Hyeon Seok Hwang, Ju-Young Moon, Kyung-Hwan Jeong, Sang-Ho Lee, So-Young Lee, Gang Jee Ko, Dong-Young Lee, Hong joo Lee, Yang Gyun Kim

**Affiliations:** 1 Division of Nephrology, Department of Internal Medicine, CHA Bundang Medical Center, CHA University, Seongnam, South Korea; 2 Division of Nephrology, Department of Internal Medicine, Kyung Hee University School of Medicine, Seoul, South Korea; 3 Department of Internal Medicine, Korea University College of Medicine, Seoul, South Korea; 4 Division of Nephrology, Department of Internal Medicine, Veterans Health Service Medical Center, Seoul, South Korea; 5 Division of Nephrology, Department of Internal Medicine, Seoul Red Cross Hospital, Seoul, South Korea

**Keywords:** Gait speed, Handgrip strength, Physical performance, Hemodialysis, Mortality

## Abstract

**Background:**

Low physical performance in patients undergoing maintenance hemodialysis is associated with a high mortality rate. We investigated the clinical relevance of gait speed and handgrip strength, the two most commonly used methods of assessing physical performance.

**Methods:**

We obtained data regarding gait speed and handgrip strength from 277 hemodialysis patients and evaluated their relationships with baseline parameters, mental health, plasma inflammatory markers, and major adverse clinical outcomes. Low physical performance was defined by the recommendations suggested by the Asian Working Group on Sarcopenia.

**Results:**

The prevalence of low gait speed and handgrip strength was 28.2 and 44.8%, respectively. Old age, low serum albumin levels, high comorbidity index score, and impaired cognitive functions were associated with low physical performance. Patients with isolated low gait speed exhibited a general trend for worse quality of life than those with isolated low handgrip strength. Gait speed and handgrip strength showed very weak correlations with different determining factors (older age, the presence of diabetes, and lower serum albumin level for low gait speed, and lower body mass index and the presence of previous cardiovascular events for low handgrip strength). Patients with low gait speed and handgrip strength had elevated levels of plasma endocan and matrix metalloproteinase-7 and the highest risks for all-cause mortality and cardiovascular events among the groups (adjusted hazard ratio of 2.72, *p* = 0.024). Elderly patients with low gait speed and handgrip strength were at the highest risk for poor clinical outcomes.

**Conclusion:**

Gait speed and handgrip strength reflected distinctive aspects of patient characteristics and the use of both factors improved the prediction of adverse clinical outcomes in hemodialysis patients. Gait speed seems to be a better indicator of poor patient outcomes than is handgrip strength.

## Background

The increasing prevalence of end-stage renal disease (ESRD) is a major public health problem in most developed countries, including South Korea [1, 2]. Despite remarkable advances in dialysis modality and patient care, the mortality rate of ESRD patients is still exceedingly high compared with that of the general population [3]. Well-established risk factors for major adverse events associated with ESRD include old age, preexisting cardiovascular disease, the presence of diabetes, and underdialysis [4–10]. Nonetheless, hemodialysis patients exhibit high interindividual variability, and it is frequently difficult to predict the clinical course accurately on an individual level. The identification and management of potential risk factors is of particular importance because individualized therapeutic interventions might improve the clinical outcomes of ESRD patients.

Sarcopenia is defined as quantitative and qualitative loss of skeletal muscle that is frequently linked to adverse effects in patients [11]. Uremic toxins in chronic kidney disease (CKD) patients are often associated with not only the chronic catabolic state of inflammation, oxidative stress, and nutritional imbalance but also a high prevalence of cardiovascular events, all of which eventually lead to clinically evident sarcopenia. Recent studies have highlighted that reduced physical performance is independently associated with poor patient survival and poor quality of life among CKD patients [12, 13], indicating the importance of physical activity in risk stratification among these patients. Currently, however, the optimal method of assessing physical performance in these populations has not yet been defined.

Measurements of gait speed (GS) and handgrip strength (HS) are used as reliable tests to determine the functioning of skeletal muscle [14, 15]. Both tests are simple, rapid, inexpensive, and can be performed in the geriatric population [16]. Accumulating evidence suggests that these parameters are useful for predicting outcomes in CKD [17–19] and ESRD patients [20–24]. Nonetheless, both tests have several limitations, such as a nonstandardized protocol or intraindividual variability. Moreover, performing either test may result in the misinterpretation of the performance status because dialysis patients frequently exhibit isolated problems in their upper or lower extremities but not the other parts of their body. Therefore, it can be speculated that combining these two simple tests may compensate for the shortcomings of each individual test. The aim of this study was to determine whether GS and HS have distinctive clinical relevance and whether combining these tests could offer a better indicator of patient outcomes than performing a single test.

## Methods

### Participant and study design

**Fig. 1:**
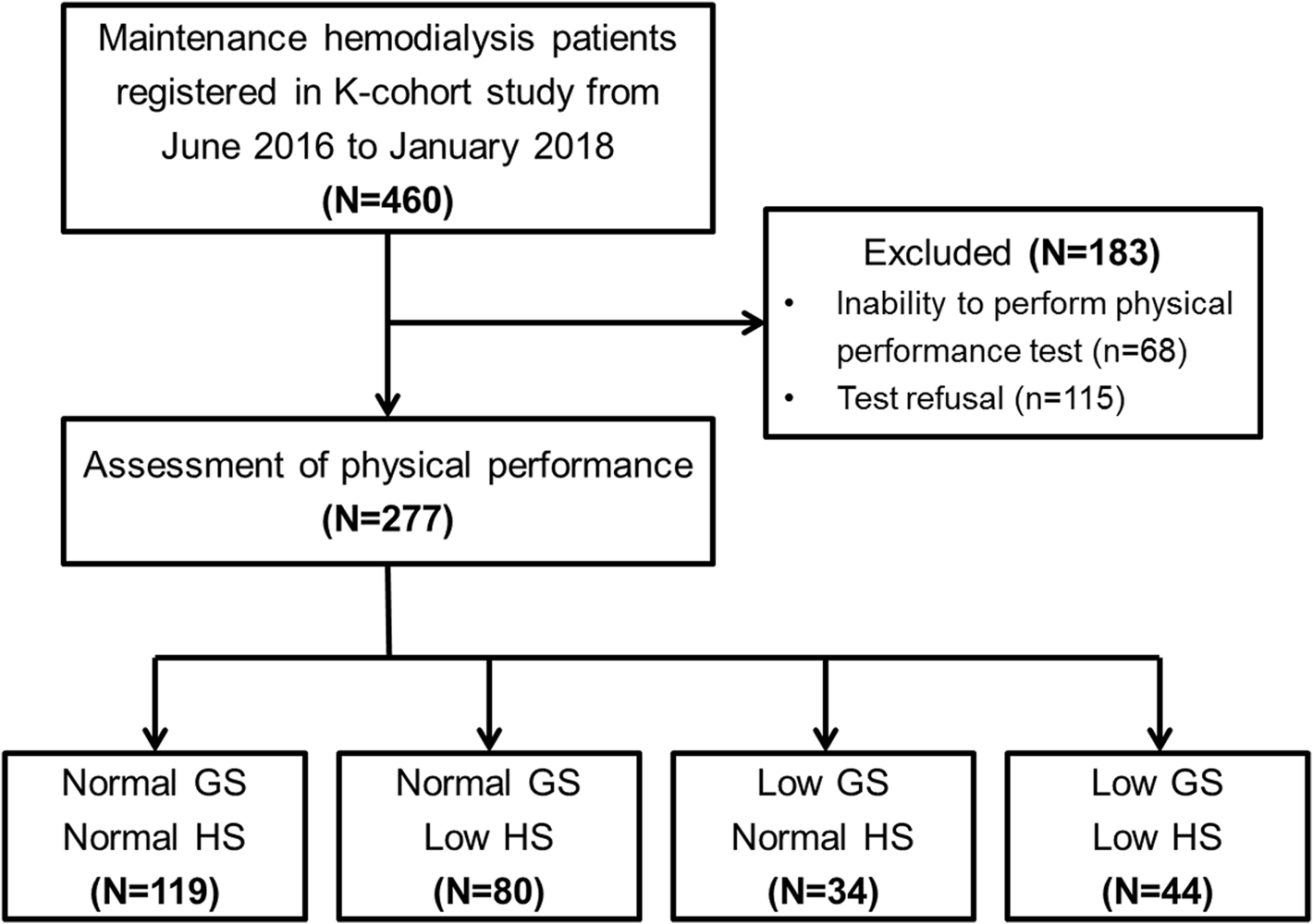
A flowchart of the study participant selection Abbreviations: GS, gait speed; HS, handgrip strength

This study was performed using the data obtained from the K-cohort, a prospective cohort of 460 hemodialysis patients who visited six hospitals between June 2016 and January 2018 (CRIS no. KCT0003281). Inclusion/exclusion criteria was described previously [25]. The patient recruitment strategy is illustrated in Fig. 1. In brief, after excluding 68 patients who were unable to be assessed for their physical performance because of their medical conditions and 115 patients who refused the tests, a total of 277 patients were finally enrolled in this study. We subsequently classified the enrolled patients into 4 groups based on their physical performance: normal GS and HS (*n* = 119, 43.0%), normal GS and low HS (*n* = 80, 28.9%), low GS and normal HS (*n* = 34, 12.3%), and low GS and HS (*n* = 44, 15.9%). Baseline demographics and clinical parameters, including the Charlson [26] and Liu [27] comorbidity indexes, were obtained at the time of study entry. All patients were monitored for major adverse events, which were defined as all-cause mortality and cardiovascular events, including acute coronary syndrome, symptomatic heart failure, cerebral infarction and hemorrhage, and peripheral artery disease, until June 2019.

### Measurements of gait speed and handgrip strength

GS was measured after the end of a dialysis session on a treatment day with a short interdialytic interval (i.e., one-day interval) within 1 month of patient enrollment. We assessed GS by measuring the walking speed over a 4-m course at the participant’s usual pace. The test was repeated three times, and the average speed was calculated. HS was measured by a Jamar hand dynamometer (Sammons Preston Inc., Bolingbrook, IL) on the dominant hand unless contraindicated during dialysis sessions. Each measurement was repeated three times, and the highest value was noted. Based on the suggestions made by the Asian Working Group for Sarcopenia [28], low GS was defined as less than 0.8 m/s, and low HS was defined as less than 26 kg for men and less than 18 kg for women.

### Questionnaires related to physical performance and mental health

Patients were asked to complete three questionnaires at the time of initial enrollment: the Korean version of the Mini-Mental Status Examination (K-MMSE) [29], the Beck Depression Inventory (BDI) [30], and the Korean version of the Kidney Disease Quality Of Life-Short Form (KDQOL-SF) [31]. We specifically obtained information regarding 11 ESRD-targeted domains on the KDQOL-SF, and these data were subsequently categorized into three components: physical, mental, and social. The physical components included the domains of physical functioning, pain, general health, and energy/fatigue. The mental components included the domains of cognitive function, sleep, and emotional well-being. Finally, the social components included work status, quality of social interaction, social support, and social function.

### Measurement of plasma inflammatory markers

Plasma samples were collected before the initiation of dialysis and stored at − 80 °C until analysis. Multiple plasma inflammatory markers were simultaneously measured by multiplex enzyme-linked immunosorbent assay as previously described [32]. We reviewed the previous literature and selected the following candidate inflammatory markers: a proliferation-inducing ligand (APRIL), B-cell activating factor (BAFF), CXCL16, endocan, endostatin, follistatin, IL-6, IL-25, IL-18, monocyte chemoattractant protein-1 (MCP-1), MCP-2, MCP-4, matrix metalloproteinase-7 (MMP-7), MMP-8, osteoprotegerin, PCSK9, receptor activator of nuclear factor-κΒ ligand (RANKL), and tumor necrosis factor-α (TNF-α).

### Statistical analysis

All statistical analyses were performed with SPSS for Windows, version 20.0 (SPSS, Chicago, IL). Baseline characteristics and clinical parameters are expressed as the means ± standard deviations (SDs) or as the numbers of patients and percentages. Analysis of variance (ANOVA) with Bonferroni post hoc analysis, chi-square test, and Fisher’s exact test were used to compare these variables, as appropriate. Non-normally distributed variables, physical performance scores, comorbidity index, and quality of life scores were described as median [first and third interquartile rage] and compared among the subgroups by the Kruskal–Wallis test with Bonferroni post hoc analysis. We used Pearson’s correlation analyses to determine the relationship between GS and HS. Multiple logistic regression analysis was used to determine the risk factors for low GS and HS. Levels of plasma inflammatory markers were expressed as box-and-whisker plots, and their comparisons were made by ANOVA with Bonferroni post hoc analysis. Finally, Kaplan-Meier curves were generated to assess the probabilities of the patient outcomes according to GS and HS, and the Cox proportional hazards model was used for further multivariate adjustments with possible confounders including age, sex, previous history of cardiovascular disease, serum albumin levels, and Charlson comorbidity index. *P* values less than 0.05 were considered to indicate statistical significance.

## Result

### Baseline clinical characteristics of patients

The baseline demographics and laboratory parameters of patients stratified by physical performance status are shown in Table 1. The prevalence of low GS and HS was 78 (28.2%) and 124 (44.8%), respectively. Patients with low GS and HS were older and had a lower body mass index and a shorter duration of dialysis than those in the other groups. The prevalence of previous cardiovascular events and diabetes was also higher in these patients. The predialysis serum albumin and creatinine levels were significantly lower in patients with poor physical performance, while spKt/V was inversely correlated with GS and HS. Mid-arm muscle circumference (MAMC) was positively correlated with GS and HS, although the statistical significance was marginal. Finally, a higher rate of the prescription of statins was observed in patients with low GS than in those with normal GS.

**Table 1:** Baseline characteristics and clinical parameters of enrolled patients according to gait speed and handgrip strength

	Normal GS and HS (*n* = 119)	Normal GS and low HS (*n* = 80)	Low GS and normal HS (*n* = 34)	Low GS and HS (*n* = 44)	*p* value
Age (year)	58.6 ± 14.3	61.5 ± 11.1	63.7 ± 10.3	68.6 ± 12.0	< 0.001^c,e^
Sex (male, %)	75 (63.0)	61 (76.2)	19 (55.9)	28 (63.6)	0.118
BMI (kg/m^2^)	23.2 ± 4.2	22.5 ± 3.3	24.5 ± 4.5	22.1 ± 3.2	0.024^e^
Time on dialysis (year)	2.8 [0.8, 5.9]	3.3 [0.8, 7.8]	1.1 [0.3, 2.6]	1.4 [0.3, 4.8]	0.005^e^
Previous cardiovascular events (n, %)	26 (21.8)	28 (35.0)	11 (32.4)	19 (43.2)	0.039^a,c^
Diabetes mellitus (n, %)	55 (46.2)	44 (55.0)	23 (67.6)	31 (70.5)	0.017^b,c^
Pre-HD SBP (mmHg)	141 ± 20	142 ± 25	143 ± 19	140 ± 21	0.949
Access type (n, %)					
Arteriovenous fistula	101 (84.9)	61 (76.2)	24 (70.6)	36 (81.8)	0.343
Arteriovenous graft	15 (12.6)	16 (20.0)	10 (29..4)	6 (13.6)	
Catheter	3 (2.5)	3 (3.8)	0 (0)	2 (4.5)	
Single-pool Kt/V	1.54 ± 0.29	1.59 ± 0.26	1.57 ± 0.29	1.68 ± 0.27	0.039^c^
Hemoglobin (g/dL)	10.6 ± 1.3	10.4 ± 1.3	10.6 ± 1.2	10.2 ± 1.3	0.417
Albumin (g/dL)	3.9 ± 0.3	3.9 ± 0.3	3.8 ± 0.4	3.7 ± 0.3	0.002^c,e^
Pre-HD BUN (mg/dL)	62.8 ± 17.8	58.4 ± 13.9	55.9 ± 17.7	54.5 ± 23.1	0.027^c^
Pre-HD creatinine (mg/dL)	9.7 ± 3.1	9.4 ± 2.2	8.1 ± 2.8	7.5 ± 2.6	< 0.001^b,c,e^
Ca (mg/dL)	8.5 ± 0.8	8.7 ± 0.8	8.3 ± 0.7	8.7 ± 0.9	0.037^d^
P (mg/dL)	5.0 ± 1.4	4.7 ± 1.2	4.7 ± 1.4	4.4 ± 1.6	0.153
Intact PTH (pg/mL)	190 [128, 302]	221 [119, 303]	185 [104, 370]	106 [58, 234]	0.060
Total CO2 (mEq/L)	22.5 ± 3.0	23.3 ± 3.1	22.6 ± 2.9	23.3 ± 2.9	0.246
β2-microglobulin (mg/L)	25.2 ± 8.6	24.4 ± 7.4	21.4 ± 7.3	25.1 ± 9.3	0.142
Total cholesterol (mg/dL)	143 ± 29	132 ± 28	145 ± 32	141 ± 31	0.019^e^
LDL cholesterol (mg/dL)	78 ± 24	73 ± 25	75 ± 26	85 ± 28	0.095
HDL cholesterol (mg/dL)	45 ± 15	45 ± 12	45 ± 16	45 ± 13	1.000
Gait speed^*^ (m/s)	1.14 ± 0.24	1.09 ± 0.18	0.66 ± 0.13	0.62 ± 0.13	< 0.001^b,c,d,e^
Handgrip strength^*^ (kg)					
Male	31.9 ± 9.2	19.4 ± 4.2	26.3 ± 13.6	18.9 ± 4.9	< 0.001^a,b,c,d,f^
Female	21.4 ± 9.6	14.2 ± 2.1	18.4 ± 6.1	13.7 ± 2.2	< 0.001^a,c^
MAMC^†^ (cm)	23.3 ± 3.4	22.5 ± 4.4	22.3 ± 2.5	21.6 ± 3.5	0.074
Anti-hypertensive medication (n, %)					
Renin-angiotensin system blocker	67 (56.3)	42 (52.5)	23 (67.6)	25 (56.8)	0.524
Calcium channel blocker	74 (62.2)	44 (55.0)	21 (61.8)	30 (68.2)	0523
β-blocker	46 (38.7)	30 (37.5)	19 (55.9)	20 (45.5)	0.250
HMG-CoA reductase inhibitor	51 (42.9)	31 (38.8)	21 (61.8)	27 (61.4)	0.022^c,d,e^

**Abbreviations**: *GS* Gait speed; *HS* Handgrip strength; *BMI* Body mass index; *HD* Hemodialysis; *CV* Cardiovascular; *SBP* Systolic blood pressure; *BUN* Blood urea nitrogen; *PTH* Parathyroid hormone; *LDL* Low-density lipoprotein; *HDL* High-density lipoprotein; *MAMC* Mid-arm muscle circumference

^*^Low GS was defined as a gait speed of less than 0.8 m/s, and low HS was defined as < 26 kg for men and < 18 kg for women

^†^MAMC was calculated by the following: MAMC = Midarm Circumference - (3.14163 Χ Triceps Skinfold Thickness / 10)

^a^*p* < 0.05, Normal GS and HS vs. Normal GS and low HS; ^b^*p* < 0.05, Normal GS and HS vs. Low GS and normal HS; ^c^*p* < 0.05, Normal GS and HS vs. Low GS and HS; ^d^*p* < 0.05, Normal GS and low HS vs. Low GS and normal HS; ^e^*p* < 0.05, Normal GS and low HS vs. Low GS and HS

Data are expressed as mean ± standard deviation or the number of patients (percentage). Time on dialysis and intact PTH levels were non-normally distributed and therefore described as median [first and third interquartile rage]

### Associations among physical performance, comorbidity index scores, and mental health

**Fig. 2:**
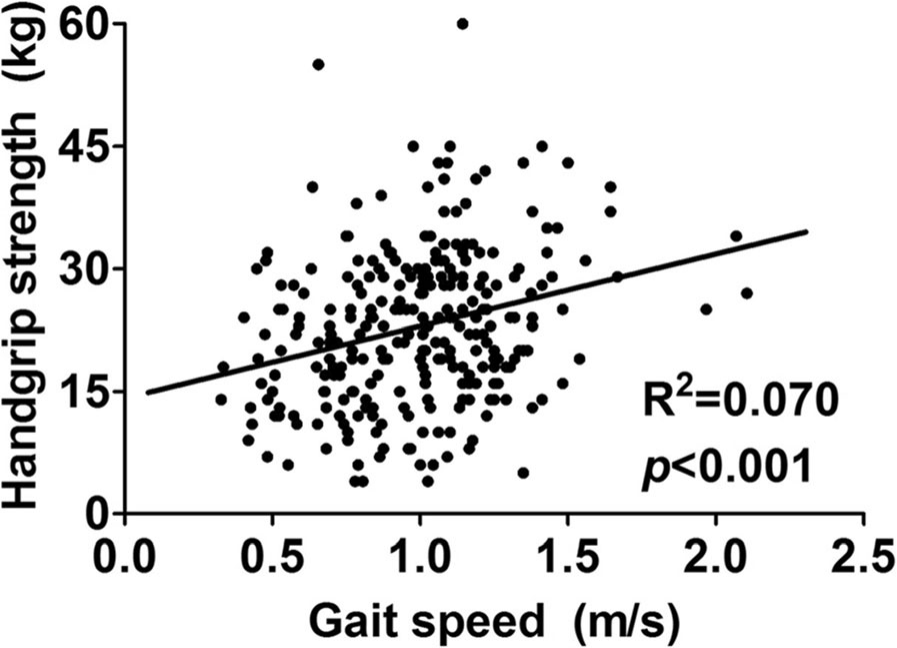
Correlation between gait speed and handgrip strength. Shown is the scatter plot displaying the relationship between gait speed and handgrip strength. Although these two parameters were significantly correlated with each other, the correlation was very weak (*R*^*2*^ = 0.070, *p* < 0.001)

We performed a correlation analysis to determine the relationship between GS and HS and found that the two parameters were significantly correlated with each other, but the correlation was weak (*R*^*2*^ = 0.070 and *p* < 0.001; Fig. 2). We next evaluated the relationships among physical performance, comorbidity index scores, and mental health. As shown in Table 2, GS and HS were significantly associated with comorbidity scores and poor physical status (Charlson comorbidity scores of 4 [2, 4] vs. 4 [3, 5] vs. 5 [3, 5] vs. 5 [4, 5] and Liu comorbidity scores of 4 [3, 5] vs. 4 [3, 6] vs. 6 [4, 7] vs. 6 [4, 7] for the normal GS and HS, normal GS and low HS, low GS and normal HS, and low GS and HS groups, respectively; *p* < 0.001 for both comparisons). In addition, patients with low GS and HS showed profoundly impaired cognitive functioning as assessed by the MMSE and the KDQOL-SF (28 [26, 29] vs. 27 [24, 28] vs. 27 [25, 30] vs. 27 [23, 29] and 87 [80, 100] vs. 87 [67, 100] vs. 80 [60, 93] vs. 73 [60, 93], normal GS and HS vs. normal GS and low HS vs. low GS and normal HS vs. low GS and HS groups; *p* = 0.030 and 0.007, respectively). The social activity index was relatively maintained in the low GS and HS groups. Notably, the comorbidity scores, depression index scores, and quality of life scores were generally worse in patients with low GS and normal HS compared to those with normal GS and low HS, although statistical significance was only observed for the physical functioning status.

**Table 2:** Association between physical performance, comorbidity index, and quality of life

	Normal GS and HS	Normal GS and low HS	Low GS and normal HS	Low GS and HS	*p* value
Charlson comorbidity scores^*^	4 [2, 4]	4 [3, 5]	5 [3, 5]	5 [4, 5]	< 0.001^a,b,c,e^
Liu comorbidity scores^*^	4 [3, 5]	4 [3, 6]	6 [4, 7]	6 [4, 7]	< 0.001^c,e^
K-MMSE^†^	28 [26, 29]	27 [24, 28]	27 [25, 30]	27 [23, 29]	0.030^c^
BDI^†^	14 [7, 21]	13 [9, 20]	17 [6, 25]	16 [11, 28]	0.122
KD-QOL^†^					
Physical components					
Physical functioning	85 [65, 95]	77 [50, 90]	60 [23, 80]	40 [25, 65]	< 0.001^b,c,d,e^
Pain	73 [58, 100]	76 [55, 100]	78 [45, 90]	55 [45, 80]	0.050^c^
General health	35 [25, 50]	40 [25, 57]	35 [21, 49]	25 [15, 35]	0.003^c,e^
Energy/fatigue	45 [35, 59]	50 [39, 55]	50 [40, 55]	40 [20, 50]	0.049^c^
Mental components					
Cognitive function	87 [80, 100]	87 [67, 100]	80 [60, 93]	73 [60, 93]	0.007^b,c^
Sleep	60 [50, 73]	60 [53, 75]	58 [46, 65]	58 [43, 65]	0.367
Emotional well-being	60 [45, 72]	60 [52, 76]	60 [52, 68]	52 [40, 64]	0.089
Social components					
Work status	0 [0, 50]	0 [0, 50]	0 [0, 50]	0 [0, 50]	0.170
Quality of social interaction	67 [60, 80]	67 [60, 87]	67 [53, 80]	67 [60, 80]	0.772
Social support	67 [67, 100]	67 [67, 100]	67 [67, 100]	67 [50, 100]	0.902
Social function	75 [63, 100]	75 [50, 100]	75 [38, 100]	63 [50, 75]	0.021^c^

**Abbreviations**: *GS* Gait speed; *HS* Handgrip strength; *K-MMSE* Korean-version of mini-mental state exam; *BDI* Beck’s depression inventory; *KD-QOL* Kidney disease quality of life

^*^Charlson comorbidity score and Liu comorbidity score are adapted from reference 25 and 26

^†^The detailed information regarding the list of questionnaire can be checked in reference 28–30

^a^*p* < 0.05, Normal GS and HS vs. Normal GS and low HS; ^b^*p* < 0.05, Normal GS and HS vs. Low GS and normal HS; ^c^*p* < 0.05, Normal GS and HS vs. Low GS and HS; ^d^*p* < 0.05, Normal GS and low HS vs. Low GS and normal HS; ^e^*p* < 0.05, Normal GS and low HS vs. Low GS and HS

### Risk factors for low gait speed and poor handgrip strength

Logistic regression analysis was performed to identify the determining factors of poor physical performance (Table 3). Older age was the only common risk factor for both low GS (adjusted odds ratio [OR] of 1.51, 95% confidence interval [CI] of 1.20–1.91; *p* < 0.001) and low HS (adjusted OR of 1.30, 95% CI of 1.07–1.57; *p* = 0.008). The presence of diabetes and low serum albumin levels were risk factors for low GS (adjusted OR of 2.12, 95% CI of 1.16–43.86 and adjusted OR of 3.37, 95% CI of 1.32–8.62, respectively) but not for low HS. On the other hand, low HS but not low GS was significantly associated with low BMI (adjusted OR of 0.92, 95% CI of 0.86–0.99; *p* = 0.022) and a previous history of cardiovascular events (adjusted OR of 1.73, 95% CI of 1.02–2.95; *p* = 0.043).

**Table 3:** Logistic regression on the determinant factors of low gait speed and low handgrip strength

	Low gait speed				Low handgrip strength			
	Univariate		Multivariate		Univariate		Multivariate	
	OR (95% CI)	*p* value	OR (95% CI)	*p* value	OR (95% CI)	*p* value	OR (95% CI)	*p* value
Age (per 10 years increment)	1.61 (1.28–2.01)	< 0.001	1.51 (1.20–1.91)	0.001	1.32 (1.10–1.59)	0.004	1.02 (1.00–1.4)	0.026
Male (vs. female)	1.42 (0.83–2.45)	0.202			0.63 (0.38–1.04)	0.072		
BMI (per 1 kg/m^2^ increment)	1.01 (0.95–1.08)	0.702			0.92 (0.86–0.98)	0.014	0.92 (0.86–0.99)	0.022
Time on dialysis (per 1 year increment)	0.91 (0.86–0.98)	0.008	0.94 (0.88–1.01)	0.081	1.02 (0.97–1.06)	0.451		
Diabetes (vs. absent)	2.27 (1.30–3.96)	0.004	2.12 (1.16–43.86)	0.014	1.47 (0.91–2.38)	0.114		
Previous cardiovascular event (vs. absent)	1.68 (0.97–2.92)	0.066			1.91 (1.14–3.21)	0.014	1.73 (1.02–2.95)	0.043
Albumin (per 1 g/dL decrement)	4.90 (2.03–11.83)	< 0.001	3.37 (1.32–8.62)	0.011	1.69 (0.79–3.62)	0.177		

**Abbreviations**; *OR* Odds ratio; *CI* Confidence interval; *BMI* Body mass index

### The relationship between plasma inflammatory markers and physical performance

**Fig. 3:**
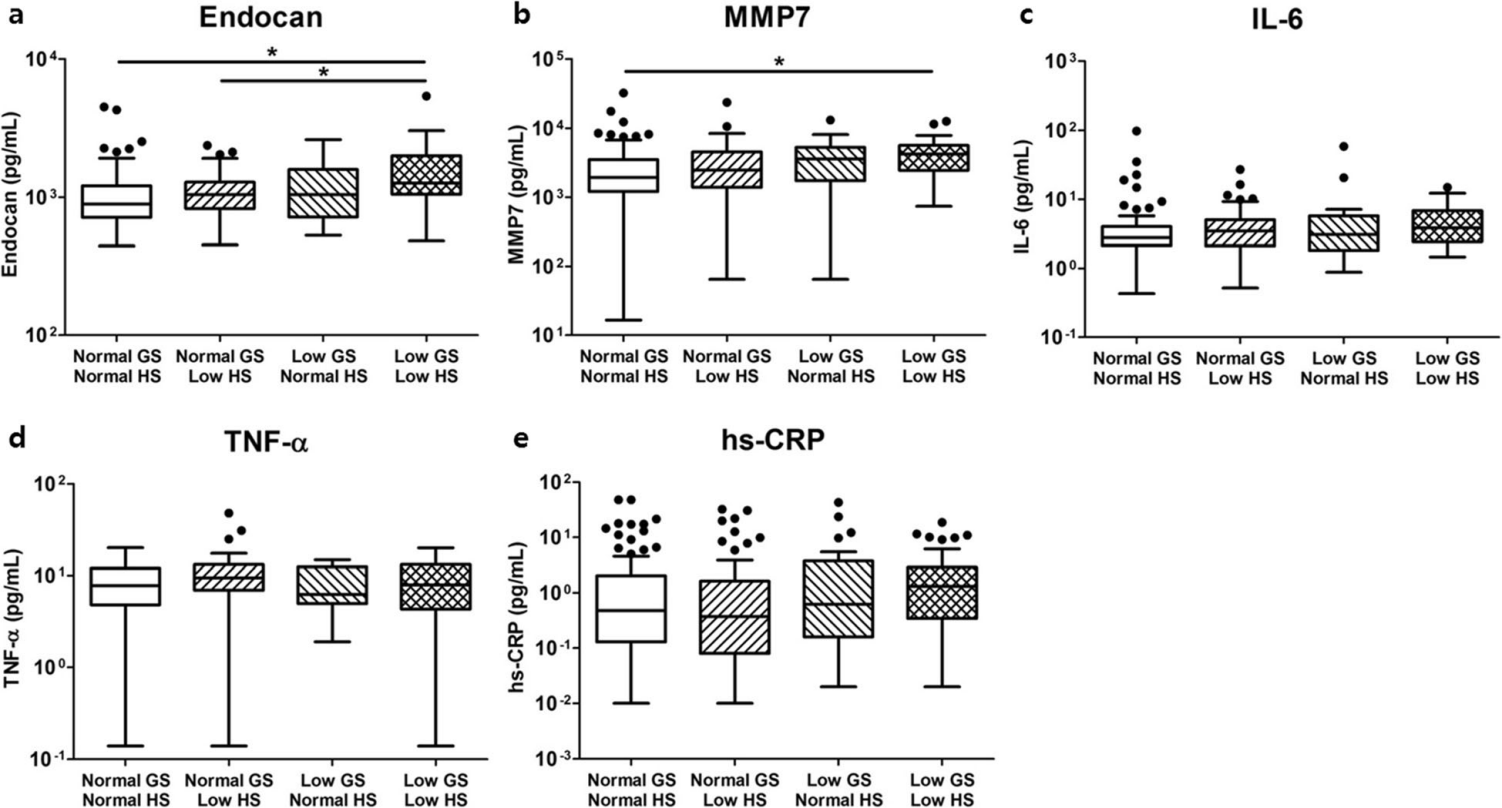
Levels of various plasma inflammatory markers in hemodialysis patients according to physical performance. Among the plasma inflammatory markers measured by multiplex enzyme-linked immunosorbent assay, the levels of (**a**) endocan and (**b**) MMP-7 were significantly higher in patients with low GS and HS than in those with normal GS and HS. The levels of (**c**) TNF-α, (**d**) IL-6, and (**e**) hs-CRP were not different among the groups. Abbreviations: GS, gait speed; HS, handgrip strength; MMP, matrix metalloproteinase; TNF, tumor necrosis factor; hs-CRP, high-sensitivity C-reactive protein

We next measured various plasma inflammatory markers and compared their levels across the groups. Among the cytokines and chemokines, the levels of plasma endocan and MMP-7 were significantly higher in patients with low GS and HS than in those with normal GS and HS (Fig. 3a and b). In contrast, the levels of traditional inflammatory markers, including TNF-α, IL-6, and high sensitivity C-reactive protein (hs-CRP), were not associated with physical performance (Fig. 3c-e).

### Impacts of gait speed and handgrip strength on all-cause mortality and cardiovascular events

**Fig. 4:**
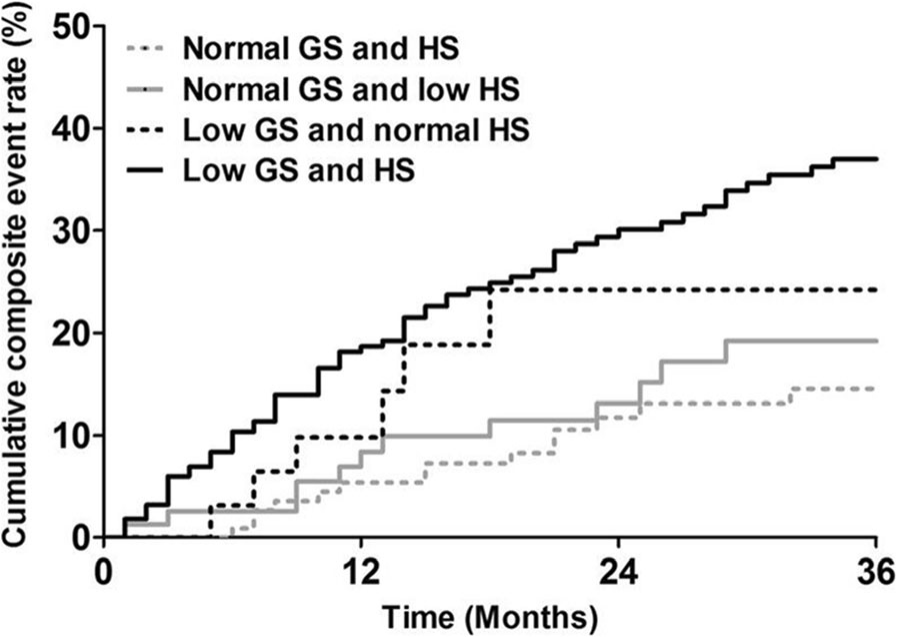
Cumulative event rate of all-cause mortality and cardiovascular events in hemodialysis patients according gait speed and handgrip strength. Patients with low GS and HS showed the highest cumulative composite event rate (*p* = 0.004 for overall trends). Abbreviations: GS, gait speed; HS, handgrip strength

The mean duration of follow-up since the recruitment of patients was 25.3 months, and a total of 19 deaths (6.9%) and 30 (10.8%) cardiovascular events occurred during this period. Patients with low GS and HS showed the highest cumulative incidence rate for major adverse events (11.8, 15.0, 17.6, and 29.5% for the normal GS and HS, normal GS and low HS, low GS and normal HS, and low GS and HS groups, respectively, *p* = 0.004 for overall comparisons; Fig. 4).

**Fig. 5:**
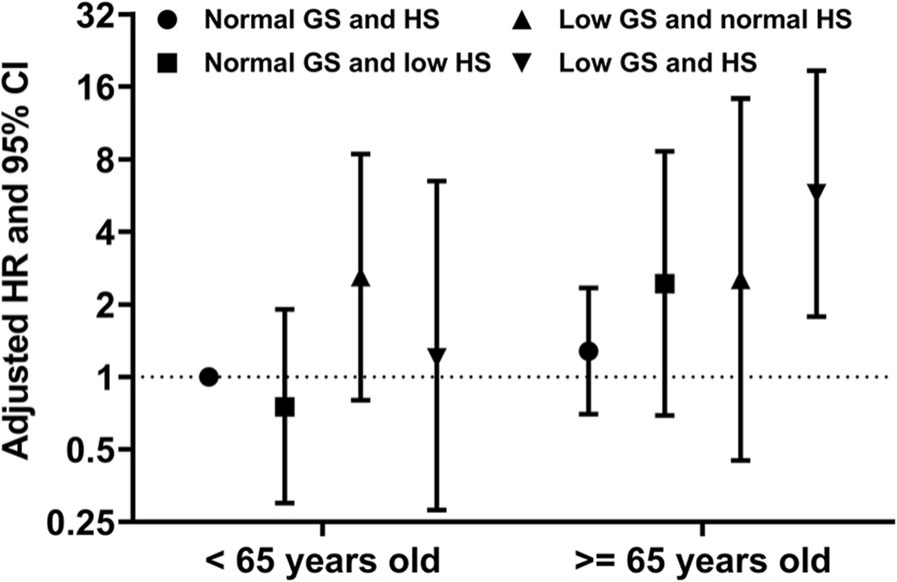
Adjusted hazard ratios of the cumulative composite event rate based on age and physical performance. Elderly hemodialysis patients with low GS and HS exhibited a significantly higher risk of major adverse events (adjusted HR of 5.76, 95% CI of 1.78–18.62; *p* = 0.012), while physical performance was not associated with composite outcomes in patients under 65 years. The outcome was defined as the composite of all-cause mortality and/or major cardiovascular events, and the composite incidence rate of hemodialysis under 65 years with normal GS and HS was set as the reference. Dots and I bars indicate adjusted HR and 95% CI, respectively. Abbreviations: GS, gait speed; HS, handgrip strength**;** HR, hazard ratio; CI, confidence interval

The observed hazard ratios (HRs) for major adverse events are shown in Table 4. Multivariate Cox regression analysis revealed that patients with low GS and HS had the highest level of risk for major adverse events (adjusted HR of 2.72, 95% CI of 1.14–6.46; *p* = 0.024) compared to the risk levels of those with normal GS and HS after multivariate adjustments of possible confounders. Patients with normal HS but low GS also exhibited a tendency toward an increase in major adverse events (adjusted HR of 2.38, 95% CI of 0.86–6.53; *p* = 0.084). In contrast, isolated low HS was not related to an increased risk of adverse outcomes, although the adjusted HRs were slightly elevated. Notably, low GS and HS was associated with significantly increased composite event rate even after adjustment with patient’ comorbidity scores (adjusted HR of 2.30, 95% CI of 1.02–5.21; *p* = 0.045). There was a significant interaction between GS and HS for major adverse events (*p* = 0.019). Finally, we performed a subgroup analysis of enrolled patients according to their age. As shown in Fig. 5, physical performance was not associated with composite outcomes in hemodialysis patients under 65 years of age. In contrast, the risk of major adverse events was significantly increased in elderly patients with low GS and HS (adjusted HR of 5.76, 95% CI of 1.78–18.62; *p* = 0.012).

**Table 4:** Incidence and hazard ratios of cumulative composite event rate based on the physical performance

	No. of events (%)	*p* for interaction	Adjustment model 1^a^ (HR [95% CI])	*p* value	Adjustment model 2^a^ (HR [95% CI])	*p* value
Cumulative composite event rate^b^						
Normal GS and HS	14 (11.8)	0.019	Reference	–	Reference	–
Normal GS and low HS	12 (15.0)		1.08 (0.49–2.39)	0.843	1.15 (0.52–2.54)	0.737
Low GS and normal HS	6 (17.6)		2.38 (0.86–6.53)	0.084	1.92 (0.69–5.31)	0.211
Low GS and HS	13 (29.5)		2.72 (1.14–6.46)	0.024	2.30 (1.02–5.21)	0.045

^a^ Model 1 was adjusted by age, sex, previous history of cardiovascular disease, and serum albumin levels. Model 2 was adjusted by Charlson comorbidity score

^b^ Cumulative incidence of all-cause mortality and cardiovascular events

**Abbreviations**: *HR* Hazard ratios; *CI* Confidence interval; *GS* Gait speed; *HS* Handgrip strength

## Discussion

Although sarcopenia was originally described as an age-related structural and functional decline in skeletal muscle, recent investigations have consistently acknowledged that decreased kidney function is also involved in sustained muscle wasting and the subsequent development of sarcopenia. Compared to the elderly population, in which the prevalence of sarcopenia is 11% [33], CKD patients are likely to be much more prone to its occurrence, with an estimated prevalence of 30–60% [20, 21, 24, 34–36]. The two main components of sarcopenia, muscle strength and mass, are dissociated in the setting of ESRD, and the muscle strength is more important than muscle mass in terms of patient outcomes [20, 35]. In line with these findings, a recent metanalysis showed a strong association between CKD progression and slowing of walking speed [37]. In this context, we extensively investigated the effects of skeletal muscle dysfunction on major adverse events in hemodialysis patients. Our findings suggest that GS and HS represent different aspects of patient characteristics and that their combination could identify those at the highest risk for mortality and cardiovascular events. Of note, MAMC showed a tendency to be relatively lower in patients with poor physical performance but was not related to either clinical outcome (data not shown). Together, our data support the idea that the functional assessment of skeletal muscle is more important than its quantitative assessment and that measuring GS and HS is a suitable method for the evaluation of skeletal muscle function in hemodialysis patients.

Based on the significant correlation between poor physical performance and high mortality in CKD patients, several prospective trials and metanalysis have assessed whether exersice intervention could improve patient outcomes [38–45]. Although physical training significantly improved patient quality of life and inflammatory parameters in most studies, these benefits were not translated into better patient survival. One of the reasons for this discrepancy might be that patients enrolled in these studies were highly heterogeneous in their baseline clinical characteristics, underlying comorbidities, and laboratory findings. Moreover, there is no consensus on the definition of adequate exercise for hemodialysis patients, thereby limiting the application of intradialytic exercise in routine clinical practice. Therefore, well-designed randomized controlled trials are needed to clarify the clinical significance of intradialytic exercise, especially in terms of improving patient mortality.

We noticed that spKt/V, currently used as a standard method for the assessment of dialysis adequacy, was highest in patients with low GS and HS and lowest in patients with normal GS and HS (Table 1). The inverse relationship between Kt/V and physical performance was consistently shown in other studies, suggesting that this relationship is likely to be a universal phenomenon [24, 34, 36, 46]. We speculate that the low muscle mass and subsequent decreased volume of distribution of urea in the body (V) in patients with low GS and HS resulted in a relative increase in the value of Kt/V without affecting the true dialysis efficacy [47]. Therefore, sarcopenic patients may be underdialyzed if their dialysis time and dialyzer filter are selected solely based on the levels of Kt/V. Further study is warranted to define the optimal target of Kt/V in dialysis patients based on the severity of sarcopenia.

Although GS and HS are the two representative tests used to assess physical performance, direct comparisons of these parameters have rarely been made, especially in dialysis patients. Here, we examined their relationship and found that a substantial portion of patients exhibited low performance on one test while demonstrating normal performance on the other (114/277, 41.2%). Moreover, the correlation coefficient between GS and HS was very weak despite its statistical significance, suggesting that the factors contributing to these two conditions might be different. We consider that this finding is at least in part due to the differences in the muscles and neurologic systems involved during the execution of the HS and GS tests. In accordance with our data, Roshanravan et al. showed a discrepancy in upper and lower muscle strength in a nondialysis CKD cohort study [19]. Thus, these data provide a rationale that the combination of the GS and HS tests could integrate the different patient components, thereby allowing us to predict future outcomes better.

Despite the fact that the clinical relevance of GS and HS as predictors of mortality and cardiovascular outcomes was documented in previous studies, direct comparisons between these two tests have not been performed so far. Interestingly, patients with isolated low GS had a tendency to exhibit worse comorbidity indexes and physical functions than those with isolated low HS (Table 2). Furthermore, GS was significantly superior than HS for the prediction of all-cause mortality in the analysis of our cohort, implying that the muscle function of the lower extremities might be more important than that of the upper extremities in terms of patient outcomes. Several recent studies also revealed that skeletal muscle function in the lower extremities but not in the upper extremities was associated with overall physical performance and the hospitalization rate [48, 49], emphasizing the clinical importance of lower extremity performance. Moreover, the GS test is still valuable because low GS is associated with increased HRs for death and cardiovascular mortality regardless of HS (Fig. 3 and Table 4). Johansen et al. investigated longitudinal trends in the physical performance of hemodialysis patients and found that GS frequently declined while HS did not change over time [50]. GS was the strongest individual predictor of future frailty and mortality among various physical activity assessment tools, including HS, which is in line with our findings. Therefore, we consider that monitoring gait functions has the potential to serve as a valuable tool for continuous risk stratification of dialysis patients.

We found that the levels of endocan and MMP-7 were elevated in patients with low GS and HS. Endocan is a water-soluble proteoglycan consisting of amino acid polymers and a single dermatan sulfate chain [51]. Plasma endocan is known to exclusively originate from the vascular endothelium, and its levels reflect endothelial activation and systemic inflammation. Several previous studies have demonstrated the clinical value of plasma endocan in the prediction of cardiovascular mortality as well as the progression of kidney diseases [52–55]. It should be confirmed whether elevated levels of plasma endocan result from sarcopenia itself or from other confounding factors, such as vascular injuries or infection [56, 57]. MMP-7 is an endopeptidase that belongs to the MMP family. In addition to its basic functions in cleaving extracellular matrix substrates, MMP-7 is also involved in the development of local and systemic inflammation [58–60]. Although MMP-2 and MMP-9 seem to play major roles in the degradation of the extracellular matrix that leads to muscle wasting, the pathophysiological relevance of MMP-7 in the development and progression of sarcopenia is still mostly unknown. Increased MMP-7 activity is observed in a hereditary form of muscular dystrophy [61], suggesting that upregulated MMP-7 might have detrimental effects on skeletal muscle. In contrast with a previous report [20], the levels of hs-CRP, IL-6, and TNF-α were not elevated in sarcopenic patients in our study. We speculate that these inconsistent findings are attributable to the differences in the degree of overall inflammation; the absolute concentrations of hs-CRP and IL-6 were lower and the levels of serum albumin were higher in patients in our study than in those in the previous study [20].

Although low GS or HS alone was not predictive of patient outcomes in our cohort (Table 4), several other studies showed that isolated low GS or low HS was an independent predictor of all-cause mortality in patients with CKD [19–22]. This discrepancy is, at least in part, because the number of cardiovascular events in this study during follow-up was low. Thus, the statistical power of multivariable analysis with respect to separately analyzing the prognostic impacts of GS and HS was reduced. Moreover, the appropriate cutoff values for low GS and HS are still controversial, even though guidelines had already been established for Asian populations [28]. More vigorous validations are needed to determine the clinical relevance of these criteria as predictors of patient outcomes.

The limitations of this study should be mentioned. There is a concern about selection bias because patients who were incapable of performing the GS and/or HS tests were excluded from our study. Indeed, a previous study reported that dialysis patients who could not complete a walking test had the highest comorbidity index and worst survival rate, even when compared to those who could walk very slowly (< 0.6 m/s) [21]. Plasma inflammatory markers were not adjusted for other clinical parameters. Thus, the impacts of these markers on patient outcomes were substantially limited. Nonetheless, we believe that these results may help clinicians assess the overall status of hemodialysis patients since their levels could reflect physical performance. Finally, we could not determine the possible mechanisms underlying the association between low physical performance and high mortality. We speculate that chronic sustained inflammation might be an essential mediator that contributes to both phenomena (Fig. 3). This hypothesis should be explored in further studies.

## Conclusion

Our data suggest that poor physical performance, as assessed by GS and HS, was significantly associated with high all-cause mortality and cardiovascular diseases in hemodialysis patients. GS and HS seem to capture the function of different sets of skeletal muscles, neurological impairments, and malnutrition that develop in ESRD patients. Given that the measurements of GS and HS are relatively easy to perform, the combination of these two tests would provide clinicians opportunities for better patient assessment and individualized care.

## Data Availability

The datasets used and/or analyzed in the current study are available from the corresponding author on reasonable request.

## Abbreviations

ESRD: End-stage renal disease
CKD: Chronic kidney disease
GS: Gait speed
HS: Handgrip strength
K-MMSE: Korean version of the mini-mental state examination
BDI: Beck depression inventory
KDQOL-SF: Kidney disease quality of life-short form
MMP: Matrix metalloproteinase
TNF: Tumor necrosis factor
MAMC: Mid-arm muscle circumference
OR: Odds ratio
CI: Confidence interval
hs-CRP: High-sensitivity C-reactive protein
HR: Hazard ratio
